# Construction of an Epithelial-Mesenchymal Transition-Related Model for Clear Cell Renal Cell Carcinoma Prognosis Prediction

**DOI:** 10.1155/2022/3780391

**Published:** 2022-08-09

**Authors:** Shimiao Zhu, Tao Wu, Ziliang Ji, Zhouliang Wu, Hao Lin, Chong Shen, Yinggui Yang, Qingyou Zheng, Hailong Hu

**Affiliations:** ^1^Department of Urology, Tianjin Institute of Urology, The Second Hospital of Tianjin Medical University, Tianjin 300211, China; ^2^Department of Urology, Shenzhen Hospital, Southern Medical University, Shenzhen 518100, China

## Abstract

**Background:**

A rising amount of data demonstrates that the epithelial-mesenchymal transition (EMT) in clear cell renal cell carcinomas (ccRCC) is connected with the advancement of the cancer. In order to understand the role of EMT in ccRCC, it is critical to integrate molecules involved in EMT into prognosis prediction. The objective of this project was to establish a prognosis prediction model using genes associated with EMT in ccRCC.

**Methods:**

We acquired the mRNA expression profiles and clinical information about ccRCC from TCGA database. In this study, we measured differentially expressed EMT-related genes (DEEGs) by two comparison groups (tumor versus normal tissues; “stages I-II” versus “stages III-IV” tumor tissues). Based on classification and regression random forest models, we identified the most important DEEGs in predicting prognosis. Afterwards, a risk-score model was created using the identified important DEEGs. The prediction ability of the risk-score model was calculated by the area under the curve (AUC). A nomogram for prognosis prediction was built using the risk-score in combination with clinical factors.

**Results:**

Among the 72 DEEGs, the classification and regression random forest models identified six hub genes (DKK1, DLX4, IL6, KCNN4, RPL22L1, and SPDEF), which exhibited the highest importance values in both models. Through the expression of these six hub genes, a novel risk-score was developed for the prognosis prediction of ccRCC. ROC curves showed the risk-score performed well in both the training (0.749) and testing (0.777) datasets. According to the survival analysis, individuals who were separated into high/low-risk groups had statistically different outcomes in terms of prognosis. Besides, the risk-score model also showed outstanding ability in assessing the progression of ccRCC after treatment. In terms of nomogram, the concordance index (C-index) was 0.79. Additionally, we predicted the differences in response to chemotherapy drugs among patients from low- and high-risk groups.

**Conclusion:**

Gene signatures related to EMT could be useful in predicting ccRCC prognosis.

## 1. Introduction

RCC accounts for 2 to 3% of all cancers worldwide [1]. Almost 403,000 people are diagnosed with RCC each year, and 175,000 people die from it [2]. There is a range of histological classification groups, but kidney renal clear cell carcinoma (KIRC, ccRCC) is the most prevalent and contributes to the majority of renal cancer-related deaths. KIRC can remain clinically occult in the absence of significant clinical symptoms, and patients are initially diagnosed when they are already at a late stage of the TNM. In general, cases of late diagnosis are associated with lower survival rates, which results in a lower five-year survival rate for KIRC patients. In stage I, the five-year disease-specific survival for RCC patients ranges from 80 to 95 percent, but it will drop to less than 10% for stage IV patients [3]. For these RCC patients who had a lower survival rate and high risk, more elaborate and customized treatment plans were necessary. As a result, prognostic models that are capable of accurately identifying patients at high risk are urgently needed.

The EMT process describes the transition of epithelial cells to mesenchymal cells in a series of steps, and it is characterized by a loss of polarity, a breakdown in the integrity barrier, and an increase in invasion [4]. Many studies have highlighted the significance of EMT in cancer metastasis and pharmaceutical resistance [5]. The abnormal EMT signature is associated with various acquired capabilities, such as resistance to chemotherapy and immunotherapy, in addition to migration and invasion [6]. Recently, an EMT signature was shown to be linked to immune cell signaling, providing novel insights into the link between EMT and immune activation [7]. There are potential therapeutic opportunities because of the association between EMT and immune cells. Although EMT-related signatures have been linked to ccRCC metastasis and prognosis, limited studies have been conducted to determine if they can be employed as indicators for early detection and prognosis assessment.

In the current study, random forest models were developed to identify the most important genes associated with KIRC patient survival time and survival status. A prognostic risk-score model for KIRC was developed by the expression of six important genes. The AUC values and survival analysis results demonstrated the feasibility and accuracy of the risk-score model. A nomogram was constructed to predict overall survival (OS) in KIRC after incorporating the risk-score and clinical data parameters. Together, our findings demonstrate the importance of risk-score and nomogram for the prediction of survival for patients with KIRC.

## 2. Materials and Methods

### 2.1. Data Collection

Level three of mRNA sequencing data of cancer patients with KIRC was collected from TCGA (https://tcga-data.nci.nih.gov/tcga/). The expression data of 539 KIRC and 72 normal kidney samples were chosen for further investigation. The form of the downloaded gene expression data was “fragments-per-kilobase-million” (FPKM). The original data was then converted into “transcript-per-million” (TPM). Among 539 KIRC samples, the numbers of stage I, stage II, stage III, and stage IV were 268, 57, 123, and 83.

### 2.2. Identification of Differentially Expressed Genes (DEGs)

The R package “edgeR” was chosen to obtain DEGs between KIRC and normal tissues [8]. The DEGs filtering criteria were established at a *p* value of less than 0.05 and a |log2FoldChange| greater than 0.5. Similarly, DEGs between early stage (“stages I-II”) and advanced stage (“stage III-IV”) tumor tissues were obtained by the same method and screening criteria. We downloaded 1184 genes related to EMT from the dbEMT online database [9], and then we obtained the DEEGs by integrating the DEGs and EMT-related genes through the R package “VennDiagram” [10].

### 2.3. Analysis of Pathways

Enrichr (https://maayanlab.cloud/enrichr/enrich) [11] was performed to identify significantly enriched pathways. Results from modules, including “GO_Biological_Process_2021,” “GO_Molecular_Function_2021,” “GO_Cellular_Component_2021,” “KEGG_2021_Human,” and “MSigDB_Hallmark_2020” were downloaded and presented in this work. Pathways with a *p* value of less than 0.05 were recognized as significant pathways.

### 2.4. Selection of Biomarkers by Machine Learning

In order to construct a model that has perfect prediction performance, we used machine learning models to select the genes that are significantly correlated with prognosis. The expression values of DEEGs were normalized by the “log2(*x* + 1)” and “min-max” normalization methods. A classification and a regression model were constructed by the random forest (RF) algorithm. The classification RF (cRF) was built for the assessment of the survival status of KIRC patients. The regression RF (rRF) was built for the prediction of the survival time of KIRC patients. The importance values of genes in two models were calculated, and the six genes with the greatest importance values were chosen for further study as hub genes.

### 2.5. Construction of the Risk Model

The expression profiles of TCGA-KIRC were separated randomly into training (70%) and testing (30%) datasets. In the training of KIRC patients, univariate Cox analysis was performed to assess the coefficients of genes. The risk-score was evaluated by the equation: risk − score = (coefficient × expression of gene 1) + (coefficient × expression of gene 2) + ⋯+(coefficient × expression of gene *X*). KIRC individuals were separated into low and high groups by the median risk-score, respectively. With the log-rank test, survival curves for low- and high-risk individuals were compared, including OS and progression-free interval events (PFI). The “survivalROC” R package was selected to calculate the AUC value to evaluate the predictive ability.

### 2.6. Stratification Analysis

TCGA-KIRC individuals were stratified into subgroups by age (≥60 years vs. <60 years), gender (female vs. male), and TNM stages (T1/T2 vs. T3/T4, N0 vs. N1, and M0 vs. M1). The “Wilcoxon rank-sum” test was selected to discover the risk-score distribution with the R package “ggpubr.”

### 2.7. Nomogram Development

A nomogram including clinical variables (age and stage) and the risk-score was designed to estimate the likelihood of one, three, and five-year OS. C-index values vary between 0.5 and 1.0, representing no discriminating ability and excellent discriminating capacity, respectively. The fit of the generated and reference lines indicates the high accuracy of the nomogram model.

### 2.8. Chemotherapeutic Response Prediction

The responses to chemotherapeutic drugs were predicted for samples by the R package “pRRophetic” [12]. With a prediction model based on Genomics of Drug Sensitivity in Cancer (GDSC) data and expression profiles of TCGA-KIRC samples, the package could predict the IC50 of each drug for each patient. The IC50 refers to the dosage required for halving the number of viable cells, and it is a measure of the drug's therapeutic effectiveness and can also be used for assessing the tolerance of tumor cells to drugs.

### 2.9. Evaluation of the Tumor Microenvironment (TME)

ESTIMATE [13] and CIBERSORT [14] were utilized in R to determine each KIRC sample's TME status. For example, ESTIMATE predicted the level of stromal, immune, and tumor is scored based on the expression profiles of TCGA-KIRC samples. The relative levels of 22 tumor-infiltrating lymphocytes (TILs) in KIRC samples were predicted by the CIBERSORT algorithm. To ensure the prediction results are credible, *p* value < 0.05 was used as the selection criterion.

## 3. Results

### 3.1. Identification of DEEGs and Functional Enrichment Analysis

**Figure 1:**
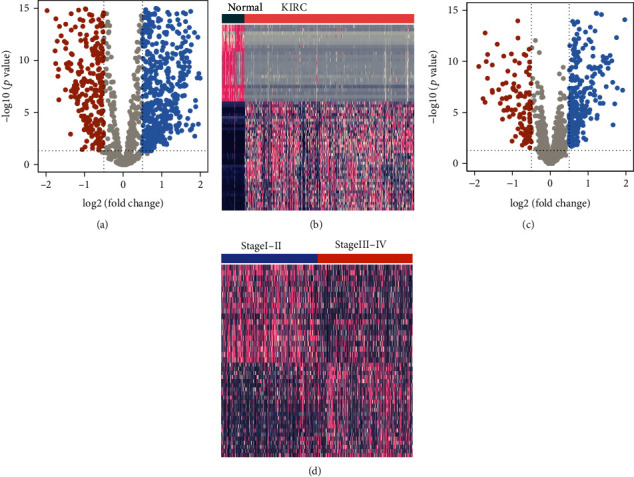
Identification of DEGs in TCGA-KIRC cohort. (a) The volcano of DEGs between KIRC and normal kidney samples. (b) The heatmap of DEGs between KIRC and normal kidney samples. (c) The volcano of DEGs between “stages I-II” and “stages III-IV” tumor tissues. (d) The heatmap of DEGs between “stages I-II” and “stages III-IV” tumor tissues. In volcano plots, red dots indicate downregulation genes in KIRC or “stages III-IV,” whereas blue dots indicate upregulation genes. In heatmap plots, red indicates high-expression values, whereas blue indicates low-expression values.

**Figure 2:**
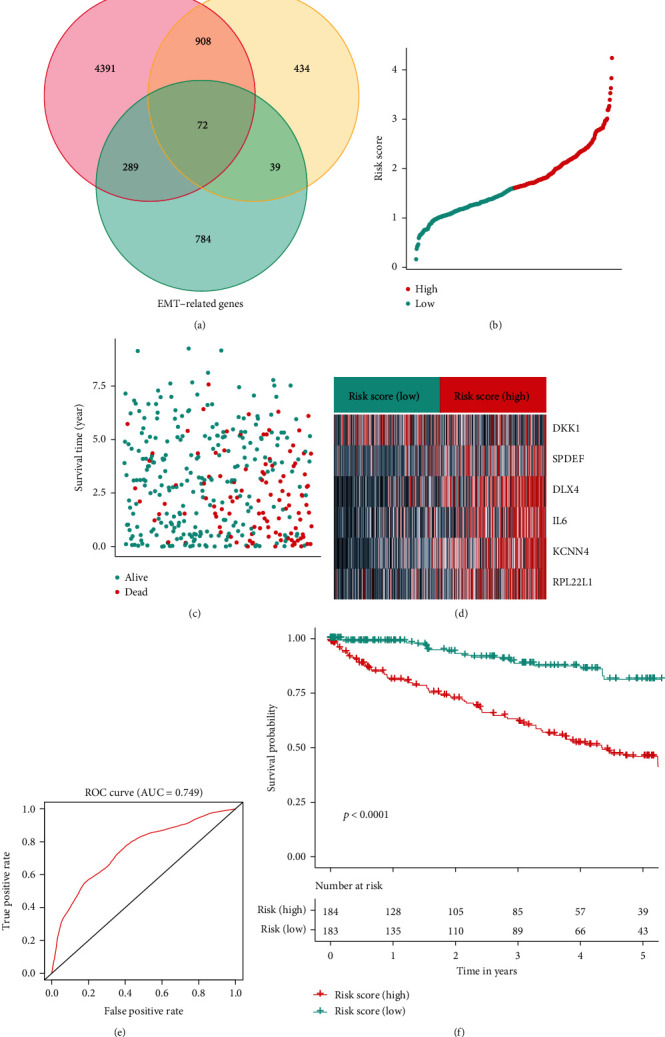
Assessment and DEEGs signature in the training dataset. (a) Intersection of DEGs and EMT-related genes by the Venn plot. (b) Risk-score distributions, (c) survival time/statuses, and (d) heatmap of the hub DEEGs expression in the training dataset. (e) The AUC value of the risk-score in the training dataset. (f) Survival curves (OS) of risk-score groups in the training dataset.

A total of 8905 significantly DEGs were identified between KIRC and normal kidney samples, of which 5660 were upregulated and 3245 were downregulated in KIRC samples than in normal samples (Figures 1(a) and 1(b)). Similarly, 2052 significantly DEGs were found between early stage (“stages I-II”) and advanced stage (“stages III-IV”) tumor tissues, of which 1453 were upregulated and 599 were downregulated in the advanced stage than in early stage KIRC samples (Figures 1(b) and 1(c)). After an intersection of EMT-related genes and DEGs by Venn diagram, 72 DEEGs were found (Figure 2(a)).

Following that, functional enrichment analysis was used to investigate the probable molecular processes behind DEEGs. The enriched biological process (BP) terms were “inflammatory_response” and “cytokine_mediated_signaling_pathway” (Supplementary Table 1). The enriched molecular function (MF) was the terms of “cytokine_activity” and “receptor_ligand_activity” (Supplementary Table 2). The significant cellular component (CC) terms were “collagen_containing_extracellular_matrix” and “secretory_granule_lumen” (Supplementary Table 3). Furthermore, the KEGG analysis indicated that DEEGs were strongly linked to pathways in “IL17_signaling” and “viral_protein_interaction_with_cytokine_and_cytokine_receptor” (Supplementary Table 4). Besides, the hallmark pathway analysis showed that “epithelial_mesenchymal_transition” and “inflammatory_response” (Supplementary Table 5).

### 3.2. Selection of EMT-Related Genes by Machine Learning Models

We built a classification and a regression model to identify the appropriate biomarkers. The classification random forest (cRF) model was built to predict the survival status (dead or alive) of KIRC patients. The importance values of genes in the cRF are shown in Table 1. Similarly, a regression random forest (rRF) model was built to predict the survival time of KIRC patients. The importance values of genes in two models were calculated (Table 1). The six genes with the highest importance values were selected as hub genes for further analysis. Among those 72 DEEGs, KCNN4, DKK1, DLX4, SPDEF, IL6, and RPL22L1 were considered hub genes since they have the highest importance values.

### 3.3. Construction of Risk-Score for KIRC

The datasets were then separated into training (70%) and testing (30%) datasets. Based on coefficients from the multivariate Cox analysis, we established the risk-score by the expression of the 6 genes by the equation: risk − score = (2.57 × KCNN4) + (0.14 × DKK1) + (1.27 × DLX4) + (1.0 × SPDEF) + (0.69 × IL6) + (0.92 × RPL22L1). The risk-score distributions, survival status, survival time, and transcriptomic levels of individuals were ordered using the risk-score (Figures 2(b)–2(d)). KIRC patients were classified as the high or low group, respectively. The AUC of the risk-score was 0.749, suggesting a high prognostic prediction ability (Figure 2(e)). According to the survival curve (OS), there was a substantial difference in OS between groups (*p* value < 0.001) (Figure 2(f)).

We then validated the 6 gene model in the testing dataset. The risk-score distributions, survival status, survival time, and transcriptomic levels of individuals were ordered using the risk-score (Supplementary Figure 1A-C). 79 and 80 KIRC individuals were classified as high or low-risk, and the AUC value was 0.777 (Supplementary Figure 1D). According to the survival curve (OS), there was a substantial difference in OS between groups (*p* value = 0.0011) (Supplementary Figure 1E).

We then validated the 6 genes to predict the progression of KIRC patients. The distributions of risk-scores, prognosis, and gene expression values of patients were ranked by risk-scores (Supplementary Figure 2A-C). 254 and 255 KIRC patients were labeled as high or low risk, respectively, and the AUC was 0.722 (Supplementary Figure 2D). Discrepancies in PFI were found between high and low groups (*p* value < 0.001) (Supplementary Figure 2E). These results suggest that our risk-score model could be an accurate indicator for OS and PFI prediction.

### 3.4. Relationship between Prognostic Signature and Clinicopathological Features

**Figure 3:**
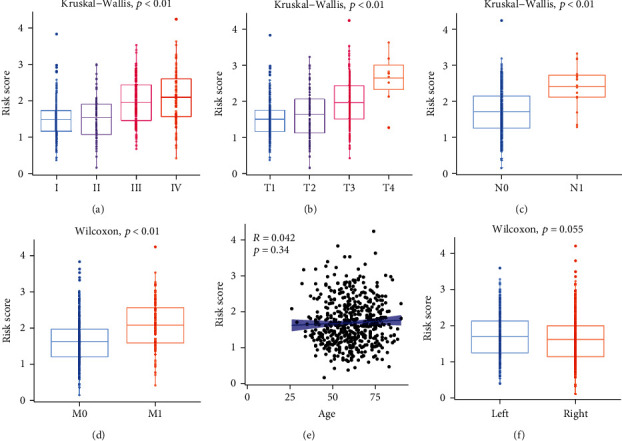
Relationship between risk-score and clinical factors, including (a) stage IV, (b) T stage, (c) N stage, (d) M stage, (e) Age, and (f) laterality.

A correlation between the prognostic signature and clinical and pathological characteristics was then examined. The results indicated a positive correlation between the risk core and poor prognosis. For example, risk-score was found in the advanced stages of KIRC, such as stage IV (Figure 3(a)), T4 (Figure 3(b)), N1 (Figure 3(c)), and M1 (Figure 3(d)). In contrast, the correlations of the risk-score with age (Figure 3(e)) and laterality (Figure 3(f)) were not significant.

### 3.5. Stratification Analysis

In the groups of “stages I-II” and “stages III-IV,” patients with higher risk had worse OS (Supplementary Figure 3A-B). Similarly, we demonstrated that risk-score could predict the OS of T1-T2 or T3-T4 patients (Supplementary Figure 3C-D), patients with TNM stage N0 (Supplementary Figure 3E), KIRC individuals with TNM stage M0 and M1 (Supplementary Figure 3G-H), patients with laterality of “left” and “right” (Supplementary Figure 3I-J), and patients with “>60” and “<60” (Supplementary Figure 3K-L). The difference in risk groups in patients with TNM stage N1 was not significant since the number of patients is low (Supplementary Figure 3F).

Afterward, we conducted the univariate/multivariate Cox regression to validate the independent prognostic role of risk-score. Univariate analysis calculated the *p* values of age (*p* value < 0.01), laterality (*p* value = 0.994), stage (*p* value < 0.01), and risk-score (*p* value < 0.01). Subsequent multivariate analysis demonstrated that age (coefficients: 0.037, *p* value < 0.01), stage (coefficients: 0.52, *p* value < 0.01), and risk-score (coefficients: 0.76, *p* value < 0.01) were negatively correlated with OS. These findings suggest that the risk-score is an independent predictor of survival in KIRC patients.

### 3.6. Construction of a Predictive Nomogram

**Figure 4:**
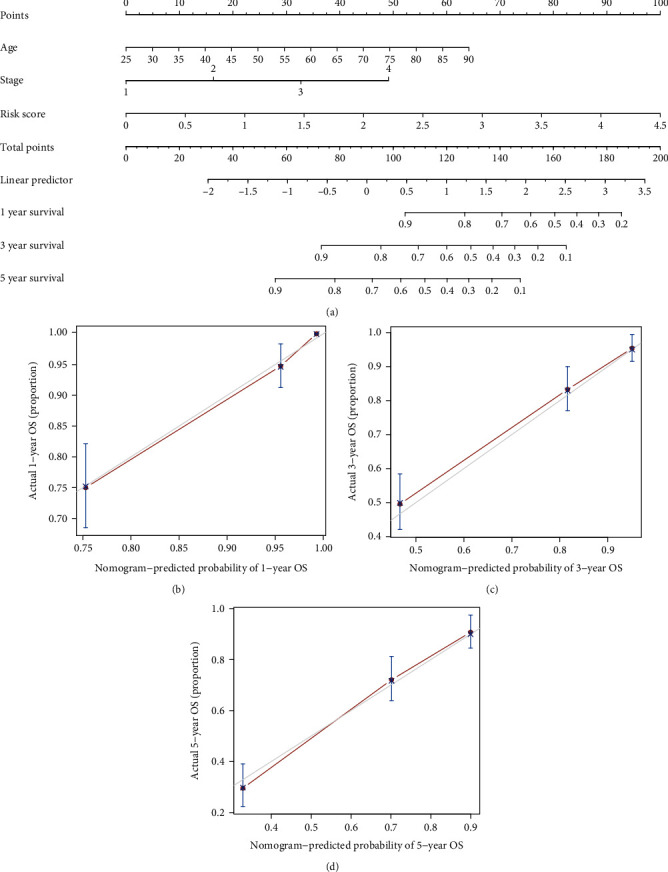
(a) The prognostic nomogram was constructed by age, stage, and risk-score. The calibration curve diagrams for (b) 1-year, (c) 3-year, and (d) 5-year had good agreement between the predicted probability and the actual probability.

By combining the risk-score and various clinical indicators, a nomogram was created to assess the survival rate (Figure 4(a)). The nomogram has a C-index of 0.79, and the risk-score clearly demonstrated greater importance than age and stage did. The prediction and reference calibration curves showed a great fit in predicting one, three, and five years of OS (Figures 4(b)–4(d)), which proves the prediction ability of the nomogram.

### 3.7. Difference in Sensitivity to Chemotherapies

**Figure 5:**
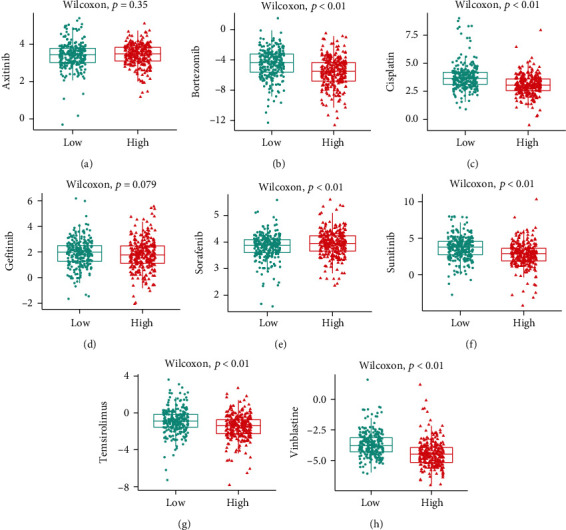
Box plot of estimated IC50 values for (a) axitinib, (b) bortezomib, (c) cisplatin, (d) gefitinib, (e) sorafenib, (f) sunitinib, (g) temsirolimus, and (h) Vinblastine in low and high risk-score groups.

The responsive predictive values of the risk-score for chemotherapy drugs (Figures 5(a)–5(h)) were calculated by IC50 values. Bortezomib, cisplatin, sunitinib, temsirolimus, and vinblastine all had lower IC50 values in the high-risk group, indicating that patients with a higher risk-score were more responsive to these medications. In the low-risk group, however, the IC50 value of sorafenib was much lower, indicating that individuals with a lower risk-score were more susceptible to it.

### 3.8. Correlation between the Risk-Score and TME

**Figure 6:**
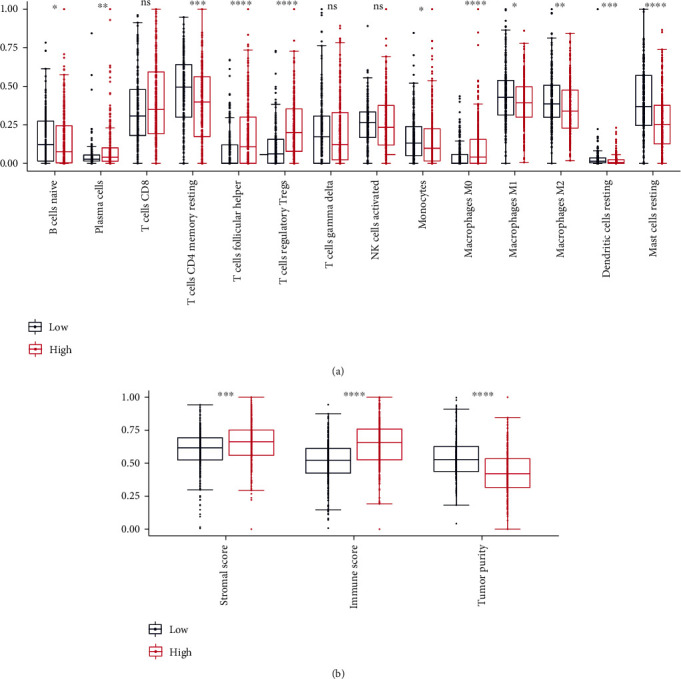
(a) Differential analysis of 14 immune fractions (CIBERSORT algorithm) between risk-score groups. (b) Differential analysis of stromal, immune, and tumor purity (ESTIMATE algorithm) between risk-score groups.

**Figure 7:**
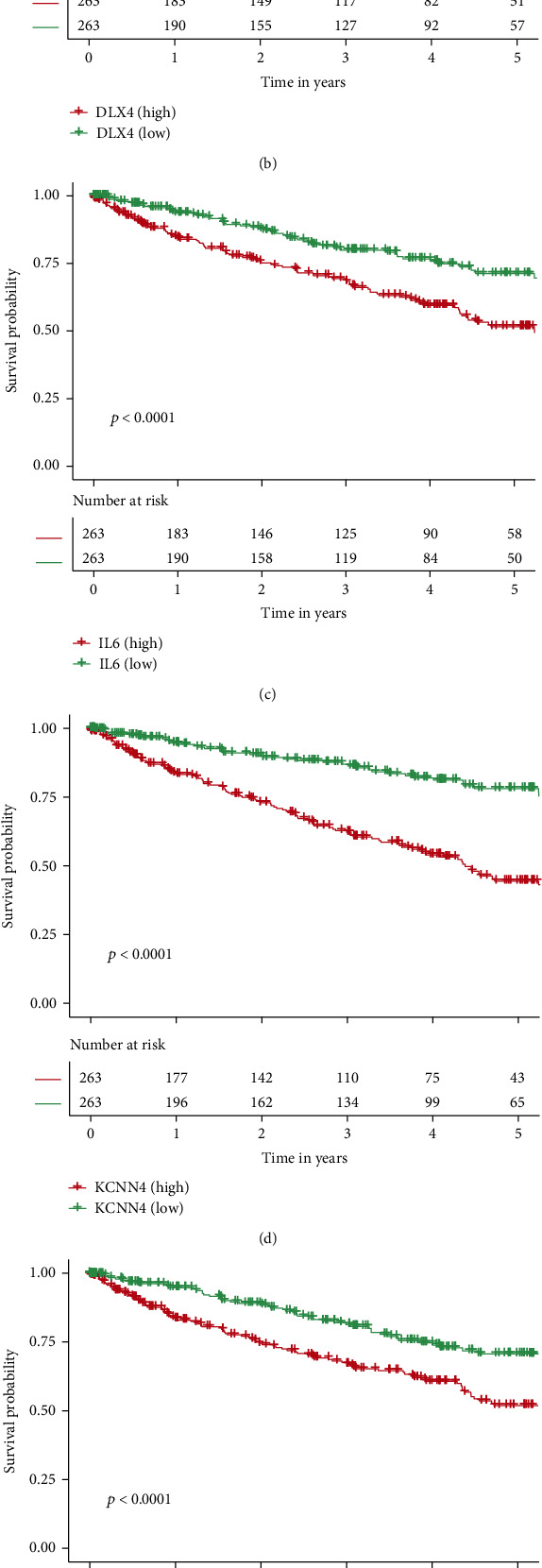
Overall survival analyses of the identified genes, including (a) DKK1, (b) DLX4, (c) IL6, (d) KCNN4, (e) RPL22L1, and (f) SPDEF in TCGA dataset. Red lines indicate patients with the high expression, whereas blue lines indicate patients with the low expression.

The CIBERSORT method was used to determine the percentage of 22 immune cells in each TCGA-KIRC sample. The cells with low mean values were deleted, and 14 cells were selected for the plot. A total of 423 samples were analyzed and found to be statistically significant. Fractions of “follicular_helper_T” and “Tregs” were higher among high-risk TCGA-KIRC samples (Figure 6(a)), while the values of “CD4_memory_T” and “NK” cells were higher among low-risk TCGA-KIRC samples (Figure 6(b)). Using the ESTIMATE technique, we also examined the differences between risk categories in terms of TME scores (Figure 6(b)). The Wilcoxon rank-sum test suggested that the immune and stromal scores in TCGA-KIRC samples were significantly higher, while the tumor purity was higher in the lower risk-score TCGA-KIRC samples. Using the Kaplan-Meier method, the prognosis of patients with higher DKK1 (Figure 7(a)) or lower DLX4 (Figure 7(b)), IL6 (Figure 7(c)), KCNN4 (Figure 7(d)), RPL22L1 (Figure 7(e)), and SPDEF (Figure 7(f)) was greatly lower.

## 4. Discussion

KIRC is particularly prone to invasion and metastasis, which may explain its poor prognosis. About 25-30% of KIRC patients have metastases at the time of diagnosis [15], and about 60% have metastases within the initial 2–3 years after diagnosis [16]. EMT is critical for tumor invasion, tumor metastasis, and tumor cell proliferation [17]. As a result, we developed a prognostic risk model for six EMT-related genes and evaluated its reliability and relationship with survival. Additionally, we checked the link between risk and response to the pharmacological therapy.

Currently, Cox regression [18] and LASSO regression [19] analyses are prevalent for identifying prognostic genes and constructing prediction models. In our study, we used machine learning models to identify the prognostic genes. Machine learning has many advantages since it can achieve a higher accuracy value with fewer genes, and it also gains the prevalence of in multiple studies [20–22]. For example, in breast cancer, a machine learning model was provided to predict the immune subtype of breast cancer [21]. The major obstacle to using machine learning models on survival data is that it contains two variables: survival status and time. Thus, we built a classification model and a regression model for predicting the survival status and time, respectively. The necessary data for these two models were the expression values of DEEGs after normalization. Based on the prediction results of these two models, we could precisely plot the survival curve for each patient. Through this method, we also successfully identified the most important genes for predicting the prognosis of KIRC.

Through the EMT process, tumors including kidney cancer could gain the potential for aggressiveness and metastasis. The activation of the EMT process is complex, but our study found that immune cells may make a significant contribution to EMT in a variety of ways. For example, some kinds of immune cells may secrete immunosuppressive molecules, hence promoting cancer progression. In our study, we discovered that Tregs were more abundant in high-risk than in low-risk samples. Tregs have been shown to impair anticancer immunity by impairing protective immunosurveillance and thwarting efficient antitumor immune responses [23]. Among high-risk samples that were linked to invasion and negative prognosis, we found that immune and stromal cells were increased but tumor purity was decreased. These results suggest that the number of immune and stromal cells might exert crucial roles in tumor development. Together, we suppose that the stromal cells and Tregs among TME increase the migration of tumor cells, which leads to a worse prognosis.

DKK1 is a Wnt signaling pathway suppressor, and its dysregulation has recently been identified as a possible biomarker for cancer development and prognosis in a variety of malignancies [24]. The amount of DKK1 expression is inversely related to the number of CD8+ T cells. DLX4, often referred to as BP1, may play a crucial role in tumor development by supporting proliferation and EMT [25]. A previous study confirmed that DLX4 contributed to the proliferation and migration of KIRC [25]. In RCC patients, high levels of interleukin-6 (IL-6) are linked to a poor prognosis [26]. IL-6 is a key diver that promotes EMT and enhances migration and invasion in KIRC tissues [27]. KCNN4 expression is higher in KIRC than in normal tissues, and its level is linked to the tumor stage and grade [28]. RPL22L1 is a ribosomal protein, and previous studies have confirmed that RPL22L1 expression is greater in cancer tissue and is linked to a worse prognosis [29, 30]. SPDEF has a complex correlation with the prognosis of cancer patients. For example, upregulation of SPDEF is associated with poor prognosis in prostate cancer [31], but it could also serve as a suppressor in colorectal cancer [32].

There are some strengths in this study. Firstly, DEEGs were derived from two comparison groups (tumor versus normal tissues; “stages I-II” versus “stages III-IV” tumor tissues) and EMT-related genes, which guarantee the clinical significance of DEEGs. Secondly, machine learning models have the ability to predict both survival time and status. Thirdly, we selected the hub DEEGs by machine learning, which increased the prediction ability of these DEEGs. For example, ROC curves showed the risk-score performed well in both the training (0.749) and testing (0.777) datasets. In terms of nomogram, the concordance index (C-index) was 0.79. Numerous limitations should be noted in our research as well. To begin with, the risk-score and nomogram were constructed using a publicly available dataset. More datasets that contain the expression data and clinical information of KIRC samples are needed to validate our results. Then, the underlying mechanisms between 6 DEEGs and KIRC progression should be clarified. Prior to clinical usage, further laboratory experiments on the six-gene signature are required.

## 5. Conclusion

In summary, EMT is critical for the advancement of cancer and is linked with worse survival in individuals with KIRC. We developed a risk-score model and a nomogram using the EMT-related genes for predicting OS in KIRC, which might enable tailored therapy and clinical decision-making for KIRC patients.

## Figures and Tables

**Table 1:** The selected hub differentially expressed EMT-related genes (DEEGs) by importance values.

Gene	Importance (cRF)	Importance (rRF)	Importance
KCNN4	57.1	80.5	137.6
DKK1	26.2	100	126.2
DLX4	73.3	52.5	125.8
SPDEF	100	18.2	118.2
IL6	49.5	65.4	114.9
RPL22L1	83.7	25.6	109.3

## Data Availability

The datasets generated for this study can be found in TCGA. Further inquiries can be directed to the corresponding authors.

## Supplementary Materials

Supplementary Materials Supplementary 1. Assessment of DEEGs signature with overall survival (OS) in testing dataset. Risk-score distributions (A), overall survival time/statuses (B), and heatmap (C) of the DEEGs expression in the testing dataset. (D) AUC values of the risk-score model in the testing dataset. (E) Kaplan-Meier estimates of OS based on the risk-score groups in the testing dataset. Supplementary 2. Assessment of risk-score model with progression-free interval (PFI). Risk-score distributions (A), PFI survival time/statuses (B), and heatmap (C) of DEEGs expression. (D) AUC values of the risk-score model. (E) Kaplan-Meier estimates of PFI based on the risk-score groups. Supplementary 3. Survival analysis of high and low risk patients in subgroups: “stages I-II” (A), “stages III-IV” (B), T1-T2 (C), T3-T4 (D), N0 (E), N1 (F), M0 (G), M1 (H), laterality of “left” (I) and “right” (J), “>60” (K), and “<60” (L). Supplementary 4. Table S1: enriched GO-BP terms from “GO Biological Process 2021” module of Enrichr webserver for all differentially expressed EMT-related genes (DEEGs). Table S2: enriched GO-MF terms from “GO Molecular Function 2021” module of Enrichr webserver for all differentially expressed EMT-related genes (DEEGs). Table S3: enriched GO-CC terms from “GO Cellular Component 2021” module of Enrichr webserver for all differentially expressed EMT-related genes (DEEGs). Table S4: enriched KEGG pathways from “KEGG 2021 Human” module of Enrichr webserver for all differentially expressed EMT-related genes (DEEGs). Table S5: enriched hallmark pathways from “MSigDB Hallmark 2020” module of Enrichr webserver for all differentially expressed EMT-related genes (DEEGs).

## References

[1] Chow W. H., Devesa S. S., Warren J. L., Fraumeni J. F. (1999). Rising incidence of renal cell cancer in the United States. *Journal of the American Medical Association*.

[2] Bray F., Ferlay J., Soerjomataram I., Siegel R. L., Torre L. A., Jemal A. (2018). Global cancer statistics 2018: GLOBOCAN estimates of incidence and mortality worldwide for 36 cancers in 185 countries. *CA: a Cancer Journal for Clinicians*.

[3] Jonasch E., Gao J., Rathmell W. K. (2014). Renal cell carcinoma. *BMJ*.

[4] Thiery J. P., Sleeman J. P. (2006). Complex networks orchestrate epithelial-mesenchymal transitions. *Nature Reviews. Molecular Cell Biology*.

[5] Ruan T., Wan J., Song Q., Chen P., Li X. (2022). Identification of a novel epithelial-mesenchymal transition-related gene signature for endometrial carcinoma prognosis. *Genes (Basel)*.

[6] De Craene B., Berx G. (2013). Regulatory networks defining EMT during cancer initiation and progression. *Nature Reviews. Cancer*.

[7] Mak M. P., Tong P., Diao L. (2016). A patient-derived, Pan-cancer EMT signature identifies global molecular alterations and immune target enrichment following epithelial-to-mesenchymal transition. *Clinical Cancer Research*.

[8] Robinson M. D., McCarthy D. J., Smyth G. K. (2010). edgeR: a bioconductor package for differential expression analysis of digital gene expression data. *Bioinformatics*.

[9] Zhao M., Liu Y., Zheng C., Qu H. (2019). dbEMT 2.0: an updated database for epithelial-mesenchymal transition genes with experimentally verified information and precalculated regulation information for cancer metastasis. *Journal of Genetics and Genomics*.

[10] Chen H., Boutros P. C. (2011). VennDiagram: a package for the generation of highly-customizable Venn and Euler diagrams in R. *BMC Bioinformatics*.

[11] Kuleshov M. V., Jones M. R., Rouillard A. D. (2016). Enrichr: a comprehensive gene set enrichment analysis web server 2016 update. *Nucleic Acids Research*.

[12] Geeleher P., Cox N., Huang R. S. (2014). pRRophetic: an R package for prediction of clinical chemotherapeutic response from tumor gene expression levels. *PLoS One*.

[13] Yoshihara K., Shahmoradgoli M., Martínez E. (2013). Inferring tumour purity and stromal and immune cell admixture from expression data. *Nature Communications*.

[14] Chen B., Khodadoust M. S., Liu C. L., Newman A. M., Alizadeh A. A. (2018). Profiling tumor infiltrating immune cells with CIBERSORT. *Methods in Molecular Biology*.

[15] Gupta K., Miller J. D., Li J. Z., Russell M. W., Charbonneau C. (2008). Epidemiologic and socioeconomic burden of metastatic renal cell carcinoma (mRCC): a literature review. *Cancer Treatment Reviews*.

[16] Mendoza-Alvarez A., Guillen-Guio B., Baez-Ortega A. (2019). Whole-exome sequencing identifies somatic mutations associated with mortality in metastatic clear cell kidney carcinoma. *Frontiers in Genetics*.

[17] Winkler J., Abisoye-Ogunniyan A., Metcalf K. J., Werb Z. (2020). Concepts of extracellular matrix remodelling in tumour progression and metastasis. *Nature Communications*.

[18] Chen Z., Liu G., Hossain A. (2019). A co-expression network for differentially expressed genes in bladder cancer and a risk score model for predicting survival. *Hereditas*.

[19] Yu S. H., Cai J. H., Chen D. L. (2021). LASSO and bioinformatics analysis in the identification of key genes for prognostic genes of gynecologic cancer. *Journal Of Personalized Medicine*.

[20] Wang Z., Chen Z., Zhao H. (2021). ISPRF: a machine learning model to predict the immune subtype of kidney cancer samples by four genes. *Translational Andrology and Urology*.

[21] Chen Z., Wang M., De Wilde R. L. (2021). A machine learning model to predict the triple negative breast cancer immune subtype. *Frontiers in Immunology*.

[22] Mohammed M., Mwambi H., Mboya I. B., Elbashir M. K., Omolo B. (2021). A stacking ensemble deep learning approach to cancer type classification based on TCGA data. *Scientific Reports*.

[23] Li C., Jiang P., Wei S., Xu X., Wang J. (2020). Regulatory T cells in tumor microenvironment: new mechanisms, potential therapeutic strategies and future prospects. *Molecular Cancer*.

[24] Chu H. Y., Chen Z., Wang L. (2021). Dickkopf-1: a promising target for cancer immunotherapy. *Frontiers in Immunology*.

[25] Sun G., Ge Y., Zhang Y. (2021). Transcription factors BARX1 and DLX4 contribute to progression of clear cell renal cell carcinoma via promoting proliferation and epithelial-mesenchymal transition. *Frontiers in Molecular Biosciences*.

[26] Wang Y., Zhang Y. (2020). Prognostic role of interleukin-6 in renal cell carcinoma: a meta-analysis. *Clinical & Translational Oncology*.

[27] Chen Q., Yang D., Zong H. (2017). Growth-induced stress enhances epithelial-mesenchymal transition induced by IL-6 in clear cell renal cell carcinoma via the Akt/GSK-3*β*/*β*-catenin signaling pathway. *Oncogenesis*.

[28] Chen S., Wang C., Su X., Dai X., Li S., Mo Z. (2021). KCNN4 is a potential prognostic marker and critical factor affecting the immune status of the tumor microenvironment in kidney renal clear cell carcinoma. *Translational Andrology and Urology*.

[29] Liang Z., Mou Q., Pan Z. (2019). Identification of candidate diagnostic and prognostic biomarkers for human prostate cancer: RPL22L1 and RPS21. *Medical Oncology*.

[30] Ma J., Jing X., Chen Z., Duan Z., Zhang Y. (2018). MiR-361-5p decreases the tumorigenicity of epithelial ovarian cancer cells by targeting at RPL22L1 and c-Met signaling. *International Journal of Clinical and Experimental Pathology*.

[31] Meiners J., Schulz K., Möller K. (2019). Upregulation of SPDEF is associated with poor prognosis in prostate cancer. *Oncology Letters*.

[32] Noah T. K., Lo Y. H., Price A. (2013). SPDEF functions as a colorectal tumor suppressor by inhibiting *β*-catenin activity. *Gastroenterology*.

