# Supplementary material for: Construction of an Epithelial-Mesenchymal Transition-Related Model for Clear Cell Renal Cell Carcinoma Prognosis Prediction
Source: Dis Markers. 2022 Aug 9;2022:3780391. doi: 10.1155/2022/3780391 (PMC9381281; doi:10.1155/2022/3780391)
Supplement: Supplementary Materials — Supplementary 1. Assessment of DEEGs signature with overall survival (OS) in testing dataset. Risk-score distributions (A), overall survival time/statuses (B), and heatmap (C) of the DEEGs expression in the testing dataset. (D) AUC values of the risk-score model in the testing dataset. (E) Kaplan-Meier estimates of OS based on the risk-score groups in the testing dataset. Supplementary 2. Assessment of risk-score model with progression-free interval (PFI). Risk-score distributions (A), PFI survival time/statuses (B), and heatmap (C) of DEEGs expression. (D) AUC values of the risk-score model. (E) Kaplan-Meier estimates of PFI based on the risk-score groups. Supplementary 3. Survival analysis of high and low risk patients in subgroups: “stages I-II” (A), “stages III-IV” (B), T1-T2 (C), T3-T4 (D), N0 (E), N1 (F), M0 (G), M1 (H), laterality of “left” (I) and “right” (J), “>60” (K), and “<60” (L). Supplementary 4. Table S1: enriched GO-BP terms from “GO Biological Process 2021” module of Enrichr webserver for all differentially expressed EMT-related genes (DEEGs). Table S2: enriched GO-MF terms from “GO Molecular Function 2021” module of Enrichr webserver for all differentially expressed EMT-related genes (DEEGs). Table S3: enriched GO-CC terms from “GO Cellular Component 2021” module of Enrichr webserver for all differentially expressed EMT-related genes (DEEGs). Table S4: enriched KEGG pathways from “KEGG 2021 Human” module of Enrichr webserver for all differentially expressed EMT-related genes (DEEGs). Table S5: enriched hallmark pathways from “MSigDB Hallmark 2020” module of Enrichr webserver for all differentially expressed EMT-related genes (DEEGs). [file 3780391.f1.zip › SupplementaryTable (1).docx]

| Pathway | Pvalue | Genes | Type |
| --- | --- | --- | --- |
| inflammatory response (GO:0006954) | <0.01 | CRP;CCL25;CXCL9;IL6;CCL20;GPR32;CCL5;LOXL3;  CXCL13;CCR5;CXCL5;S100A8 | BP |
| cytokine-mediated signaling pathway (GO:0019221) | <0.01 | CCL25;CXCL9;CSF2;EPO;MMP1;CCL20;TWIST1;IL27;CXCL13;  MMP9;CXCL5;IL6;CCL5;BIRC5;TIMP1;CCR5;JAK3 | BP |
| positive regulation of cell population proliferation (GO:0008284) | <0.01 | CSF2;EPO;SPHK1;LEF1;HMGA2;FOXM1;CXCL5;PGF;PTHLH;  IL6;TERT;MDK;CCL5;BIRC5;TIMP1 | BP |
| positive regulation of cellular process (GO:0048522) | <0.01 | CSF2;EPO;SPHK1;LEF1;HMGA2;FOXM1;DKK1;CXCL5;PGF;  PTHLH;IL6;MDK;CCL5;BIRC5;TIMP1;S100A8 | BP |
| regulation of angiogenesis (GO:0045765) | <0.01 | IL6;SPHK1;SERPINF1;SERPINE1;ADAM12;  VASH2;TWIST1;HMGA2;CXCL13;PGF | BP |
| neutrophil chemotaxis (GO:0030593) | <0.01 | CCL25;CXCL9;CCL20;CCL5;CXCL13;CXCL5;S100A8 | BP |
| granulocyte chemotaxis (GO:0071621) | <0.01 | CCL25;CXCL9;CCL20;CCL5;CXCL13;CXCL5;S100A8 | BP |
| positive regulation of angiogenesis (GO:0045766) | <0.01 | MDK;SPHK1;SERPINE1;ADAM12;VASH2;TWIST1;HMGA2;PGF | BP |
| neutrophil migration (GO:1990266) | <0.01 | CCL25;CXCL9;CCL20;CCL5;CXCL13;CXCL5;S100A8 | BP |
| cellular response to cytokine stimulus (GO:0071345) | <0.01 | CCL25;CSF2;MMP1;CCL20;LEF1;TWIST1;MMP9;IL6;  CCL5;BIRC5;TIMP1;CCR5;JAK3 | BP |

**Supplementary Table1.** Enriched GO-BP terms from “GO Biological Process 2021” module of Enrcihr webserver for all differentially expressed EMT-related genes (DEEGs).

| cytokine activity (GO:0005125) | <0.01 | CCL25;CXCL9;IL6;CSF2;EPO;CCL20;CCL5;TIMP1;CXCL13;CXCL5 | MF |
| --- | --- | --- | --- |
| receptor ligand activity (GO:0048018) | <0.01 | CCL25;CXCL9;IL6;CSF2;EPO;MDK;CCL5;TIMP1;CXCL13;DKK1;PGF;PTHLH | MF |
| chemokine activity (GO:0008009) | <0.01 | CCL25;CXCL9;CCL20;CCL5;CXCL13;CXCL5 | MF |
| chemokine receptor binding (GO:0042379) | <0.01 | CCL25;CXCL9;CCL20;CCL5;CXCL13;CXCL5 | MF |
| cytokine receptor binding (GO:0005126) | <0.01 | CCL25;IL6;EPO;CCL5;IL27;PGF | MF |
| CCR chemokine receptor binding (GO:0048020) | <0.01 | CCL25;CCL20;CCL5;CXCL13 | MF |
| CXCR chemokine receptor binding (GO:0045236) | <0.01 | CXCL9;CXCL13;CXCL5 | MF |
| sequence-specific DNA binding (GO:0043565) | <0.01 | PROP1;ONECUT2;MSX2;DLX4;TERT;UHRF1;LEF1;PRRX2;MYBL2;HOXA13;SPDEF | MF |
| metallopeptidase activity (GO:0008237) | <0.01 | MMP13;MMP1;ADAM12;MMP8;MMP9 | MF |
| double-stranded DNA binding (GO:0003690) | <0.01 | PROP1;ONECUT2;MSX2;DLX4;UHRF1;LEF1;PRRX2;MYBL2;HOXA13;SPDEF | MF |

**Supplementary Table2.** Enriched GO-MF terms from “GO Molecular Function 2021” module of Enrcihr webserver for all differentially expressed EMT-related genes (DEEGs).

| Pathway | Pvalue | Genes | Type |
| --- | --- | --- | --- |
| collagen-containing extracellular matrix (GO:0062023) | <0.01 | MDK;SERPINF1;SERPINE1;COL8A2;MMP8;  MMP9;S100A8;CTHRC1 | CC |
| secretory granule lumen (GO:0034774) | <0.01 | PLAC8;PCSK1;LRG1;SERPINE1;TIMP1;MMP8;S100A8 | CC |
| tertiary granule lumen (GO:1904724) | <0.01 | LRG1;MMP8;MMP9 | CC |
| intracellular organelle lumen (GO:0070013) | <0.01 | IL6;LRG1;MUC16;PLAUR;COL8A2;IL27;TIMP1;MMP8;MMP9 | CC |
| endoplasmic reticulum lumen (GO:0005788) | <0.01 | IL6;PLAUR;COL8A2;IL27;TIMP1 | CC |
| cyclin A2-CDK2 complex (GO:0097124) | <0.01 | CCNA2 | CC |
| specific granule (GO:0042581) | <0.01 | LRG1;PLAUR;MMP8 | CC |
| chromosome (GO:0005694) | <0.01 | BIRC5;HMGA2;HOXA13 | CC |
| spindle microtubule (GO:0005876) | <0.01 | BIRC5;AURKA | CC |
| specific granule lumen (GO:0035580) | <0.01 | LRG1;MMP8 | CC |

**Supplementary Table3.** Enriched GO-CC terms from “GO Cellular Component 2021” module of Enrcihr webserver for all differentially expressed EMT-related genes (DEEGs).

| Pathway | Pvalue | Genes | Type |
| --- | --- | --- | --- |
| IL-17 signaling pathway | <0.01 | IL6;CSF2;MMP13;MMP1;CCL20;MMP9;CXCL5;S100A8 | KEGG |
| Viral protein interaction with cytokine and cytokine receptor | <0.01 | CCL25;CXCL9;IL6;CCL20;CCL5;CXCL13;CCR5;CXCL5 | KEGG |
| Cytokine-cytokine receptor interaction | <0.01 | CCL25;CXCL9;IL6;CSF2;EPO;CCL20;  CCL5;IL27;CXCL13;CCR5;CXCL5 | KEGG |
| Pathways in cancer | <0.01 | CCNA2;IL6;TERT;EPO;MMP1;CDKN2A;LEF1;  BIRC5;PIM2;JAK3;MMP9;PGF | KEGG |
| Chemokine signaling pathway | <0.01 | CCL25;CXCL9;CCL20;CCL5;CXCL13;CCR5;JAK3;CXCL5 | KEGG |
| Rheumatoid arthritis | <0.01 | IL6;CSF2;MMP1;CCL20;CCL5;CXCL5 | KEGG |
| TNF signaling pathway | <0.01 | IL6;CSF2;CCL20;CCL5;MMP9;CXCL5 | KEGG |
| Human T-cell leukemia virus 1 infection | <0.01 | CCNA2;IL6;CSF2;MSX2;TERT;CDKN2A;JAK3 | KEGG |
| Cellular senescence | <0.01 | CCNA2;IL6;CDKN2A;SERPINE1;MYBL2;FOXM1 | KEGG |

**Supplementary Table4.** Enriched KEGG pathways from “KEGG 2021 Human” module of Enrcihr webserver for all differentially expressed EMT-related genes (DEEGs).

| Pathway | Pvalue | Genes | Type |
| --- | --- | --- | --- |
| Epithelial Mesenchymal Transition | <0.01 | PRRX1;MMP1;SERPINE1;PLAUR;DKK1;PTHLH;IL6;LOX;  ADAM12;CDH11;COL8A2;TIMP1;CTHRC1 | hallmark |
| Inflammatory Response | <0.01 | CXCL9;IL6;CCL20;SPHK1;CCL5;SERPINE1;AQP9;PLAUR;TIMP1 | hallmark |
| Allograft Rejection | <0.01 | CXCL9;IL6;CDKN2A;CCL5;TIMP1;CXCL13;CCR5;MMP9 | hallmark |
| TNF-alpha Signaling via NF-kB | <0.01 | IL6;CSF2;CCL20;SPHK1;CCL5;SERPINE1;PLAUR | hallmark |
| Complement | <0.01 | IL6;MMP13;CCL5;SERPINE1;PLAUR;TIMP1;MMP8 | hallmark |
| Hypoxia | <0.01 | PLAC8;IL6;LOX;SERPINE1;PLAUR;PGF | hallmark |
| KRAS Signaling Up | <0.01 | CSF2;PRRX1;CCL20;PLAUR;KCNN4;MMP9 | hallmark |
| Coagulation | <0.01 | MMP1;SERPINE1;TIMP1;MMP8;MMP9 | hallmark |

**Supplementary Table5.** Enriched hallmark pathways from “MSigDB Hallmark 2020” module of Enrcihr webserver for all differentially expressed EMT-related genes (DEEGs).
