# Supplementary figures and images for: Construction of an Epithelial-Mesenchymal Transition-Related Model for Clear Cell Renal Cell Carcinoma Prognosis Prediction
Source: Dis Markers. 2022 Aug 9;2022:3780391. doi: 10.1155/2022/3780391 (PMC9381281; doi:10.1155/2022/3780391)

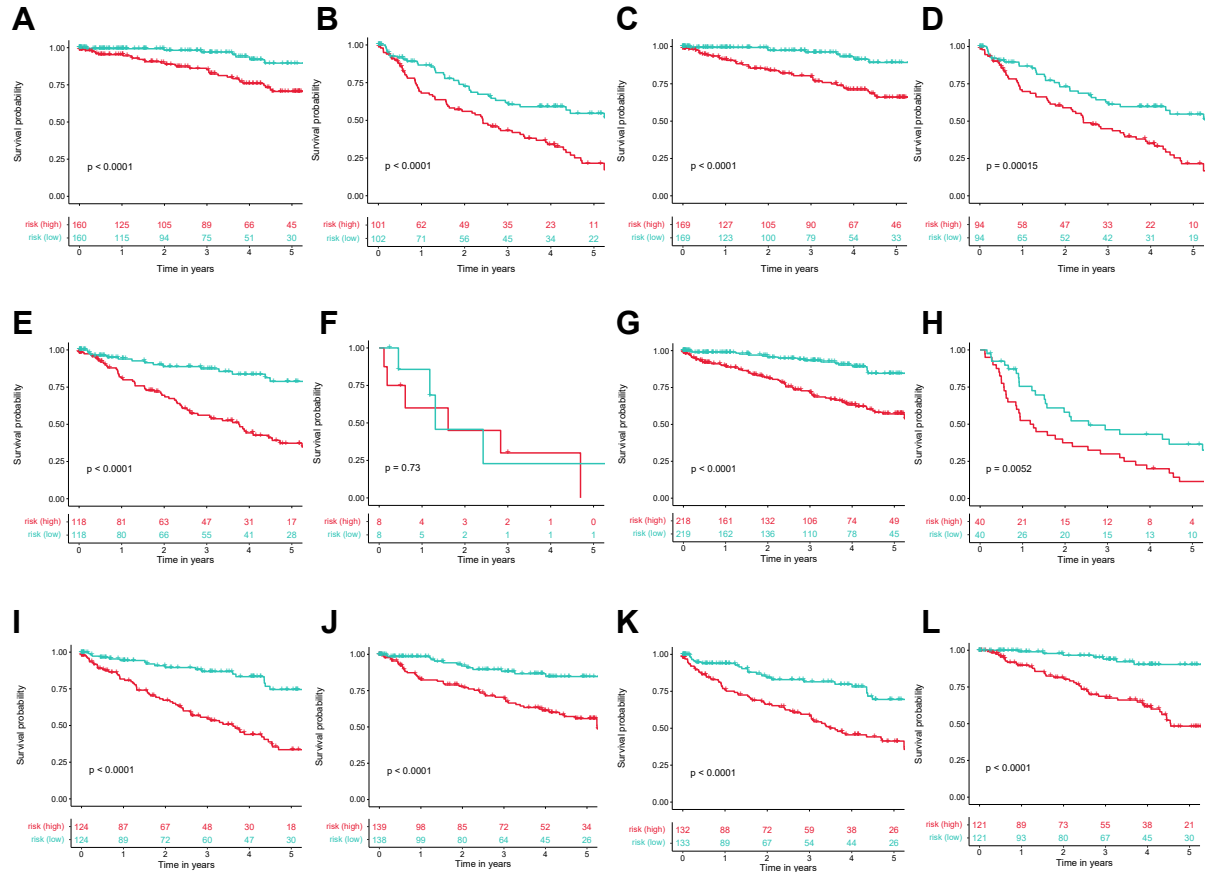

Supplement: Supplementary Materials — Supplementary 1. Assessment of DEEGs signature with overall survival (OS) in testing dataset. Risk-score distributions (A), overall survival time/statuses (B), and heatmap (C) of the DEEGs expression in the testing dataset. (D) AUC values of the risk-score model in the testing dataset. (E) Kaplan-Meier estimates of OS based on the risk-score groups in the testing dataset. Supplementary 2. Assessment of risk-score model with progression-free interval (PFI). Risk-score distributions (A), PFI survival time/statuses (B), and heatmap (C) of DEEGs expression. (D) AUC values of the risk-score model. (E) Kaplan-Meier estimates of PFI based on the risk-score groups. Supplementary 3. Survival analysis of high and low risk patients in subgroups: “stages I-II” (A), “stages III-IV” (B), T1-T2 (C), T3-T4 (D), N0 (E), N1 (F), M0 (G), M1 (H), laterality of “left” (I) and “right” (J), “>60” (K), and “<60” (L). Supplementary 4. Table S1: enriched GO-BP terms from “GO Biological Process 2021” module of Enrichr webserver for all differentially expressed EMT-related genes (DEEGs). Table S2: enriched GO-MF terms from “GO Molecular Function 2021” module of Enrichr webserver for all differentially expressed EMT-related genes (DEEGs). Table S3: enriched GO-CC terms from “GO Cellular Component 2021” module of Enrichr webserver for all differentially expressed EMT-related genes (DEEGs). Table S4: enriched KEGG pathways from “KEGG 2021 Human” module of Enrichr webserver for all differentially expressed EMT-related genes (DEEGs). Table S5: enriched hallmark pathways from “MSigDB Hallmark 2020” module of Enrichr webserver for all differentially expressed EMT-related genes (DEEGs). [file 3780391.f1.zip › SupplementaryFigure3 (1).pdf]

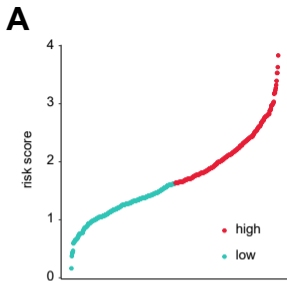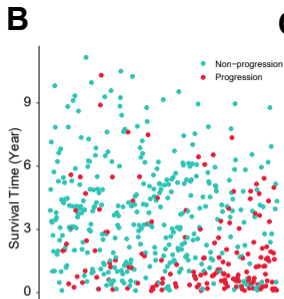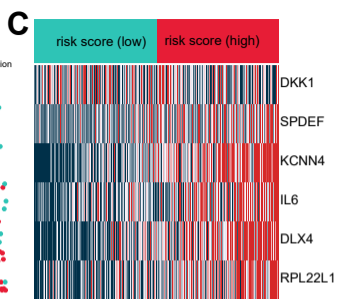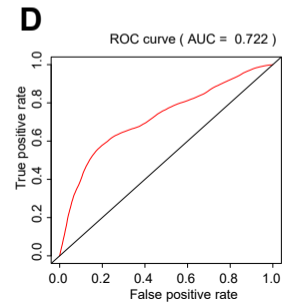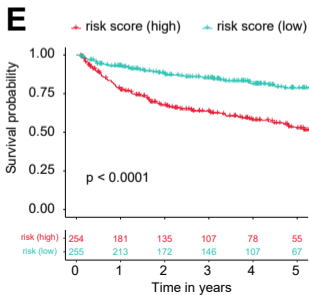

Supplement: Supplementary Materials — Supplementary 1. Assessment of DEEGs signature with overall survival (OS) in testing dataset. Risk-score distributions (A), overall survival time/statuses (B), and heatmap (C) of the DEEGs expression in the testing dataset. (D) AUC values of the risk-score model in the testing dataset. (E) Kaplan-Meier estimates of OS based on the risk-score groups in the testing dataset. Supplementary 2. Assessment of risk-score model with progression-free interval (PFI). Risk-score distributions (A), PFI survival time/statuses (B), and heatmap (C) of DEEGs expression. (D) AUC values of the risk-score model. (E) Kaplan-Meier estimates of PFI based on the risk-score groups. Supplementary 3. Survival analysis of high and low risk patients in subgroups: “stages I-II” (A), “stages III-IV” (B), T1-T2 (C), T3-T4 (D), N0 (E), N1 (F), M0 (G), M1 (H), laterality of “left” (I) and “right” (J), “>60” (K), and “<60” (L). Supplementary 4. Table S1: enriched GO-BP terms from “GO Biological Process 2021” module of Enrichr webserver for all differentially expressed EMT-related genes (DEEGs). Table S2: enriched GO-MF terms from “GO Molecular Function 2021” module of Enrichr webserver for all differentially expressed EMT-related genes (DEEGs). Table S3: enriched GO-CC terms from “GO Cellular Component 2021” module of Enrichr webserver for all differentially expressed EMT-related genes (DEEGs). Table S4: enriched KEGG pathways from “KEGG 2021 Human” module of Enrichr webserver for all differentially expressed EMT-related genes (DEEGs). Table S5: enriched hallmark pathways from “MSigDB Hallmark 2020” module of Enrichr webserver for all differentially expressed EMT-related genes (DEEGs). [file 3780391.f1.zip › SupplementaryFigure2 (1).pdf]

**A**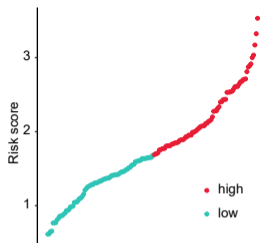**B**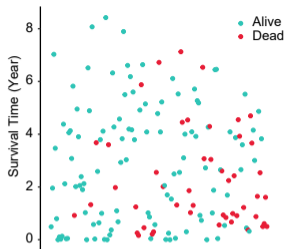**C**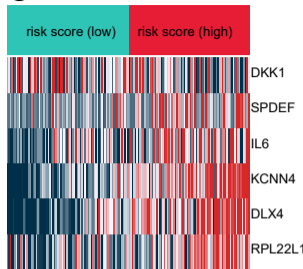**D**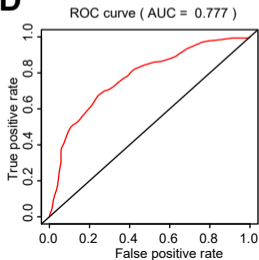**E**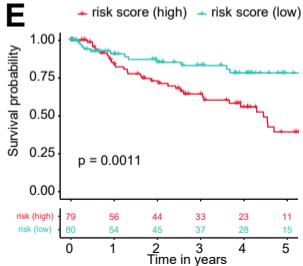

Supplement: Supplementary Materials — Supplementary 1. Assessment of DEEGs signature with overall survival (OS) in testing dataset. Risk-score distributions (A), overall survival time/statuses (B), and heatmap (C) of the DEEGs expression in the testing dataset. (D) AUC values of the risk-score model in the testing dataset. (E) Kaplan-Meier estimates of OS based on the risk-score groups in the testing dataset. Supplementary 2. Assessment of risk-score model with progression-free interval (PFI). Risk-score distributions (A), PFI survival time/statuses (B), and heatmap (C) of DEEGs expression. (D) AUC values of the risk-score model. (E) Kaplan-Meier estimates of PFI based on the risk-score groups. Supplementary 3. Survival analysis of high and low risk patients in subgroups: “stages I-II” (A), “stages III-IV” (B), T1-T2 (C), T3-T4 (D), N0 (E), N1 (F), M0 (G), M1 (H), laterality of “left” (I) and “right” (J), “>60” (K), and “<60” (L). Supplementary 4. Table S1: enriched GO-BP terms from “GO Biological Process 2021” module of Enrichr webserver for all differentially expressed EMT-related genes (DEEGs). Table S2: enriched GO-MF terms from “GO Molecular Function 2021” module of Enrichr webserver for all differentially expressed EMT-related genes (DEEGs). Table S3: enriched GO-CC terms from “GO Cellular Component 2021” module of Enrichr webserver for all differentially expressed EMT-related genes (DEEGs). Table S4: enriched KEGG pathways from “KEGG 2021 Human” module of Enrichr webserver for all differentially expressed EMT-related genes (DEEGs). Table S5: enriched hallmark pathways from “MSigDB Hallmark 2020” module of Enrichr webserver for all differentially expressed EMT-related genes (DEEGs). [file 3780391.f1.zip › SupplementaryFigure1 (1).pdf]
